# Supplementary material for: Polyketide synthase gene clusters in the ericoid mycorrhizal fungus Oidiodendron maius: genome-wide prediction, expression profiling, and functional characterization of knockout mutants
Source: Front Plant Sci. 2026 Sep 16;17:1924430. doi: 10.3389/fpls.2026.1924430 (PMC13623903; doi:10.3389/fpls.2026.1924430)
Supplement: Supplementary file 1 [file SupplementaryFile1.zip › Supplementary Materials/Supplementary Folder 1.zip!/Oidma1_AssemblyScaffolds_Repeatmasked/knownclusterblast/cluster30/jgi.p_Oidma1_105608_mibig_hits.html]

| MIBiG Protein | Description | MIBiG Cluster | MiBiG Product | % ID | % Coverage | BLAST Score | E-value |
| --- | --- | --- | --- | --- | --- | --- | --- |
| AFX60333.1 | flavin-dependent\_monooxygenase | BGC0001032 | Oocydin\_A\_biosynthetic\_gene\_cluster | 28 | 64.1509433962 | 124 | 3.6e-28 |
| AFX60310.1 | flavin-dependent\_monooxygenase | BGC0001031 | Oocydin\_A\_biosynthetic\_gene\_cluster | 28 | 64.1509433962 | 123 | 1e-27 |
| CAA11511.1 | hypothetical\_protein | BGC0000584 | Colicin\_V\_biosynthetic\_gene\_cluster | 28 | 42.0754716981 | 104 | 2.9e-22 |
